# Antioxidant Bio-Compounds from Chestnut Waste: A Value-Adding and Food Sustainability Strategy

**DOI:** 10.3390/foods14010042

**Published:** 2024-12-27

**Authors:** Roberta Barletta, Alfonso Trezza, Andrea Bernini, Lia Millucci, Michela Geminiani, Annalisa Santucci

**Affiliations:** 1 Department of Biotechnology, Chemistry and Pharmacy, University of Siena, Via Aldo Moro, 53100 Siena, Italy; r.barletta@student.unisi.it (R.B.); alfonso.trezza2@unisi.it (A.T.); andrea.bernini@unisi.it (A.B.); lia.millucci@gmail.com (L.M.); geminiani2@unisi.it (M.G.); 2 SienabioACTIVE, Università di Siena, Via Aldo Moro, 53100 Siena, Italy

**Keywords:** circular bioeconomy, antioxidant activity, bioactive compounds, waste, oxidative stress, food innovation, sustainability, docking simulation, NMR

## Abstract

In an era of escalating environmental challenges, converting organic residues into high-value bioactive compounds provides a sustainable way to reduce waste and enhance resource efficiency. This study explores the potential of the circular bioeconomy through the valorization of agricultural byproducts, with a focus on the antioxidant properties of specific chestnut burr cultivars. Currently, over one-third of food production is wasted, contributing to both humanitarian and environmental crises. Through circular bioeconomy, we can transform biological waste into valuable products for use in fields like food innovation and sustainability. The antioxidant effects of three chestnut cultivars, *Bastarda Rossa*, *Cecio*, and *Marroni*, were assessed through in vitro assays, highlighting their potential to combat oxidative stress—an important factor for health-related applications. The characterization of the three cultivars showed the major presence of ellagic acid and gallic acid in the extract, renowned for their antioxidant activity. In vitro assays evaluated the phenolic and flavonoid content, as well as the antioxidant activity of the three extracts, confirming the cultivar *Cecio* as the richest in these bioactive compounds and the most performative in antioxidant assays. In vitro antioxidant and oxidative stress recovery assays on SaOS-2, fibroblast, and chondrocyte cell lines displayed a strong antioxidant activity. Furthermore, the cytotoxicity assay demonstrated the safety of all three extracts in the tested human cell lines. In silico docking simulations further validated the biological relevance of these compounds by predicting strong hydrophobic and polar interactions with oxidative stress-related protein targets. Overall, this study demonstrates the antioxidant properties of chestnut byproducts. The findings contribute to the development of functional foods, nutraceuticals, and other applications, underscoring the role of chestnut cultivars in advancing circular bioeconomy practices.

## 1. Introduction

The chestnut tree, considered for timber and fruit, has been seeing a notable increase in global production in the last years. Italy is the European Union’s largest chestnut producer and contributes 38% of the European chestnut supply, with an annual yield of approximately 52,356 tons (FAOSTAT, accessed 18 October 2024). Chestnut cultivation has long been fundamental to Italian agriculture, especially in the mountainous regions where it once served as both a primary food source and an economic staple. In Tuscany, chestnut trees—easily recognizable by their serrated leaves and characteristic flowers—grow in specific areas, such as Piancastagnaio in Siena, which became synonymous with chestnut cultivation. These trees, which thrive in the distinctive microclimates of Mount Amiata, supported local livelihoods for generations, reinforcing the cultural and economic identity of the region. Numerous chestnut genotypes and cultivars were cultivated to meet both local and broader economic needs, increasing their significance to the communities surrounding Mount Amiata.

In addition to the historical significance, chestnut is long recognized for its health benefits, particularly in Chinese traditional medicine and more recent applications in cosmetics in Korea [1]. These uses mirror the chestnut’s bioactive potential as chestnut-derived byproducts are known to contain bioactive compounds, including phenolics, that provide health benefits through their antioxidant and radical-scavenging properties [2,3,4,5].

Variability in the phytochemical concentration of chestnut fruits, influenced by the cultivar and growth conditions, underscores the importance of selective breeding to optimize yield and nutritional quality for functional food applications [6]. Such efforts aligned with the rising trend of utilizing natural sources—flavonoids, polyphenols, and other phytochemicals—as replacements for synthetic additives, particularly in the food and nutraceutical industries. These natural antioxidants are renowned for their broad spectrum of health benefits, which include anti-cancer, anti-inflammatory, antimicrobial, and cardioprotective effects [7,8,9].

In recent years, the growing environmental crisis caused by pollution and the waste of natural resources has become a major global concern. The increasing generation of biological waste, particularly agricultural residues, exacerbates this issue. However, these byproducts offer an untapped potential for sustainable applications. Specifically, plant-based waste, such as chestnut peels [10], is rich in bioactive compounds that can be harnessed for a variety of purposes, including antimicrobial and antioxidant uses. Despite the potential, there remains a gap in research regarding the effective repurposing of such waste materials into valuable, eco-friendly products that can address both environmental and health challenges. By investigating the antioxidant properties of *Castanea sativa* peel extracts, this study aims to contribute to closing this gap, providing an innovative and sustainable solution to the dual problem of waste management and public health.

The chestnut processing industry generates approximately 7000 tons of processed chestnut products annually, producing significant waste, with burrs and shells accounting for roughly 10% of the total chestnut weight. Historically, the management of chestnut burrs involved burning them to clear the land for optimal tree growth, a practice that, although effective in the short term, is costly, time-consuming, and environmentally detrimental due to CO_2_ emissions [10].

The urgent need for more sustainable and economically viable approaches led to a growing interest in the valorization of these byproducts. Burrs are rich in tannins, ellagic acid, gallic acid, and other antioxidant biomolecules, which demonstrate significant antimicrobial, anti-carcinogenic, and cardioprotective properties [6,10,11,12,13].

This strategy followed the broader setting of the circular bioeconomy, which presents a valid alternative to the wasteful practices of the linear economy. While the linear economic model ultimately led to global resource depletion and contributed to environmental crises, the circular economy focuses on sustainable practices, encouraging resource reuse and recycling. In the case of chestnut burrs, rather than being discarded through dangerous practices like burning, these byproducts can be repurposed as valuable bioactive compounds for a variety of applications, from nutraceuticals to natural preservatives in food. This transition from a linear to a circular model is not only a matter of environmental sustainability but also offers the potential for economic growth, especially within the agri-food sector.

In fact, the circular bioeconomy has already made significant inroads in several industries. Food waste, once relegated to animal feed or discarded altogether, is now increasingly being seen as a valuable source of bioactive compounds [14,15]. Biorefineries, for example, are exploring ways to convert food waste into renewable energy, while the textile industry began utilizing natural dyes, such as those extracted from pomegranate seeds, to replace synthetic alternatives [16,17]. In the case of chestnut byproducts, including burrs, research revealed their potential to support the development of sustainable products such as natural antioxidants, antimicrobials, and even materials for use in textiles or biodegradable plastics. These bioactive compounds have the potential to be incorporated into food products, helping to combat oxidative damage, which is linked to chronic diseases like cancer, cardiovascular disease, and neurodegenerative disorders [5,9]. These extracts can also benefit the nutraceutical industry, particularly in supplements designed to address oxidative stress and inflammation. Moreover, their antimicrobial properties position chestnut extracts as valuable components for functional foods that promote gut health and food preservation, offering natural alternatives to synthetic preservatives [7,18,19,20,21]. Additionally, the bioactive compounds in chestnut extracts could be used in skincare products, offering protection from oxidative stress caused by pollutants and UV radiation [22,23].

Thus, this research addresses the critical knowledge gap on how to repurpose chestnut waste, particularly burrs, within circular bioeconomy, to yield value-added products with biological applications. By tackling the dual challenges of waste management and resource valorization, this study contributes to the development of sustainable solutions in the food and nutraceutical sectors.

As the world is facing dramatic environmental, economic, and social challenges, driven in part by the unsustainable practices of the linear economy, the circular economy offers a transformative solution [18,19]. By rethinking how natural resources, particularly agricultural byproducts, are used and valued, it is possible to create more sustainable, resilient, and resource-efficient systems. In this context, chestnut burrs exemplify the potential of agro-industrial waste to contribute to the circular bioeconomy, providing both environmental and economic benefits while offering innovative solutions for the food and health sectors [1,2].

This study aimed to dig into value-added applications for chestnut waste in the food and nutraceutical sectors. We analyzed and compared the chemical composition of hydroalcoholic extracts from three domestic cultivars certified under *Indicazione Geografica Protetta* (PGI) from Mount Amiata: *Bastarda Rossa* (RB), *Cecio* (RC), and *Marroni* (RM). These extracts showed significant phenolic contents, and ellagic and gallic acid were detected as the two most abundant compounds in all three extracts (mostly RC). Given the deep-sitting antioxidant properties of polyphenols, the total and cellular antioxidant capacities of these extracts were evaluated in vitro, along with their cytotoxicity on fibroblast, chondrocyte, and SaOS-2 cell lines. These were selected as highly relevant models: fibroblasts are critical in studying tissue repair [20,24], chondrocytes are involved in cartilage degeneration linked to oxidative damage [21,25], and SaOS-2 cells provide insights into oxidative stress in bone health [26].

All extracts demonstrated safety for cell lines and enhanced cellular recovery from oxidative stress, particularly RC and RB, which exhibited over a 50% recovery rate. The in vitro findings were supported by docking simulations, which indicated that the compounds could spontaneously bind to their predicted targets, initiating strong hydrophobic and polar interactions with key residues of the target.

Together, these findings underscore the potential of chestnut waste as a source of bioactive compounds for food innovation, and for nutraceutical development, advancing the chestnut sector’s role in sustainable food production and supporting bioeconomy goals.

## 2. Materials and Methods

### 2.1. C. sativa Shells

The shells from *C. sativa* Mill were sourced from the PGI (Protected Geographical Indication) Castagna del Monte Amiata area, a renowned chestnut-producing region in Italy. These shells, from the *Bastarda Rossa*, *Cecio*, and *Marroni* cultivars, were directly peeled from the plant. Following peeling, the shells were collected, weighed (to determine fresh weight), dried, and weighed again to determine dry weight. After drying at room temperature, the samples were homogenized using a laboratory mill and stored in dark, sealed plastic bags at −80 °C until extraction.

### 2.2. C. Sativa Shell Extract Preparation

The extraction process was carried out as follows: 100 mL of a 70/30 ethanol/water solution (*v*/*v*) was combined with 10 g of chestnut burr powder (1:10 solid-to-solid ratio). This solvent mixture was chosen for its efficiency in extracting phenolic compounds from chestnut burrs. Ethanol (Sigma-Aldrich, St. Louis, MO, USA), being polar, dissolves a wide range of bioactive compounds, including polyphenols and tannins, while the addition of water enhances the solvent’s penetration and increases the yield of these compounds. The 1/10 *w*/*v* ratio was selected to optimize the extraction of phenolics and tannins, ensuring a sufficiently concentrated extract without sacrificing practicality [27,28]. A thermostatic mantle stirred the mixture for 3 h at 80 °C. The extraction chamber was connected at the top to a net water-cooled bubble condenser, to allow the reflux of the evaporated solvent. Afterward, the mixture was centrifuged (30 min, 4000 rpm), and the supernatant was separated and filtered. The organic solvent was removed using a rotavapor, and the aqueous residue was freeze-dried to obtain the dry extract.

### 2.3. Total Phenolic Content (TPC)

The total phenolic content (TPC) was determined using the method introduced by Folin and Denis in 1912 and later modified by Folin and Ciocalteu in 1927 [29]. A calibration curve was established with gallic acid (GA) (Sigma-Aldrich, St. Louis, MO, USA) solutions. CSB samples were diluted from a stock solution (1 mg/mL) in milli-Q water. Standard and sample solutions were mixed with 1 mL of 1N FC reagent (Sigma-Aldrich, St. Louis, MO, USA) and incubated for 3 min. Subsequently, 1 mL of Na2CO3 (Sigma-Aldrich, St. Louis, MO, USA) and 7 mL of milli-Q water were added. The tubes were incubated for 90 min at room temperature, shielded from light. Absorbance was measured at 725 nm, with a blank solution serving as a control. The TPC was expressed as milligrams of GA equivalent (GAE) per 100 g of fresh material.

### 2.4. Total Flavonoid Content (TFC)

TFC was measured using the NaNO_2_-Al(NO_3_)_3_ method with slight modifications [30]. In brief, 1 mL of a sample was combined with 0.3 mL of 5% NaNO_2_ (Sigma-Aldrich, St. Louis, MO, USA) solution, and after 6 min, 0.3 mL of 10% Al(NO_3_)_3_ (Sigma-Aldrich, St. Louis, MO, USA) and 4 mL of 4% NaOH (Sigma-Aldrichm St. Louis, MO, USA) were added. The total volume was adjusted to 10 mL with distilled water, and the solution was gently shaken. Absorbance was read at 418 nm. The calibration curve was constructed using quercetin (Sigma-Aldrich, St. Louis, MO, USA), and the TFC was expressed as Quercetin Equivalents (QEs) per 100 g of fresh material.

### 2.5. Antioxidant Activity Evaluation

#### 2.5.1. FRAP Assay

The antioxidant capacity was determined using the FRAP (Ferric Ion-Reducing Antioxidant Power) assay with minor modifications [31]. An aliquot of 1 mL containing different extract concentrations (25–400 µg) was mixed with 2.5 mL of 0.2 M phosphate buffer (pH = 6.6) and 1% potassium ferricyanide solution (Sigma-Aldrich, St. Louis, MO, USA). The mixture was incubated at 50 °C for 20 min. Afterward, 2.5 mL of 10% trichloroacetic acid (Sigma-Aldrich, St. Louis, MO, USA) and 2.5 mL of distilled water were added, followed by 0.5 mL of 0.1% ferric chloride solution (Sigma-Aldrich, St. Louis, MO, USA). Absorbance was recorded at 700 nm. Results were expressed as Ascorbic Acid Equivalent (AAE) per 100 g of fresh material.

#### 2.5.2. DPPH Free Radical-Scavenging Activity

DPPH free radical-scavenging activity was assessed by mixing the extract solution with a freshly prepared DPPH (Sigma-Aldrich, St. Louis, MO, USA) solution [32]. The mixture was incubated for 20 min in the dark at room temperature, and absorbance was measured at 517 nm. Ascorbic acid (Sigma-Aldrich, St. Louis, MO, USA) was used for calibration. Radical-scavenging activity (RSA) was calculated using the formula %RSA = 100 × (A0 − As)/A0, where As is the absorbance of the sample and A0 is the absorbance of the control solution.

### 2.6. NMR Analysis

NMR samples were prepared by dissolving 50.0 mg of a dry extract in 0.5 mL of DMSO-d6 (Cambridge Isotope Laboratories, Inc., Tewksbury, MA, USA). TSP (3-(trimethylsilyl)propionic-2,2,3,3-d4 acid, Cambridge Isotope Laboratories, Inc., Tewksbury, MA, USA) was incorporated as an internal standard, reaching a final concentration of 0.3 mM. The spectra were recorded using a Bruker Advance III 600 spectrometer operating at 14.1 T. Metabolite quantification was conducted with the deconvolution feature of Chenomx 10 (Edmonton, AB, Canada). For 1H NMR, an NOESY 1D pulse sequence was employed with a 100 ms mixing time and a spectral width of 9600 Hz, digitized over 16K points. A total of 256 transients were collected with a 9 s repetition delay. Peak assignments for gallic and ellagic acids were confirmed by spiking in pure substances (Merck KGaA, Darmstadt, Germany). For 13C NMR, a 1D pulse sequence with WALTZ16 decoupling was used, covering a spectral width of 40,000 Hz and digitized over 64K points. A total of 4096 transients were accumulated with a repetition delay of 9 s, with the DMSO peak as the reference.

### 2.7. Cell Line Isolation and Culture

Primary human fibroblasts (available in our laboratory) were sourced from femoral cartilage fragments of arthropathy patients undergoing hip replacement surgery. Informed consent was obtained from the patients, and the study was approved by the Local Ethics Committee, following the guidelines of the Declaration of Helsinki (64th, 2013). Chondrocytes were isolated promptly post-surgery as previously outlined [33]. Cartilage was minced under sterile conditions and subjected to three rounds of enzymatic digestion: 1 mg/mL hyaluronidase (Sigma-Aldrich, St. Louis, MO, USA) (30 min), 5 mg/mL pronase (Sigma-Aldrich, St. Louis, MO, USA) (1 h), and 2 mg/mL collagenase (Type I, Sigma-Aldrich (St. Louis, MO, USA, 1 h) at 37 °C. The resulting cell suspension was filtered twice through 70 μm nylon meshes, washed, and centrifuged at 1500 rpm for 10 min. The pellet was resuspended in Dulbecco’s Modified Eagle’s Medium (DMEM) (Sigma-Aldrich, St. Louis, MO, USA), supplemented with 2% penicillin/streptomycin (P/S) (Sigma-Aldrich, St. Louis, MO, USA) and 10% fetal bovine serum (FBS) (Sigma-Aldrich, St. Louis, MO, USA), and seeded into 10 cm Petri dishes. Cells were cultured at 37 °C in a humidified atmosphere with 95% air and 5% CO_2_.

Primary human chondrocytes (from our laboratory) were collected from four male individuals (ages 42–50) undergoing abdominoplasty. The study was approved by the Local Ethics Committee and was conducted according to the principles of the Declaration of Helsinki (64th, 2013). Immediately after surgery, skin samples were excised and cut into 1–2 mm pieces (explants), which were placed in washing DMEM containing 10 times the usual antibiotic/antimycotic dose, plus gentamicin. These explants were minced using iris scissors, centrifuged at 200 rpm for 10 min to remove the supernatant, and then incubated with a collagenase solution (DMEM + 10% collagenase type II + 10× antibiotics/antimycotics) in a 60 mm dish at 37 °C with 5% CO_2_ for 21 h. Afterward, the explants were washed twice with PBS. For the next 1–3 days, the explants were cultured in a dish with an explant medium containing 20% heat-inactivated FBS and the usual antibiotic dose. Once collagenase was removed, the cultures were observed daily under a microscope to monitor cell migration around the explants. The explants were then transferred to a culture flask.

Once the fibroblast cultures reached confluence, they were expanded further. The medium was discarded, and the cultures were washed three times with PBS. In the second and third passages, keratinocytes were removed by brief trypsinization (0.1% trypsin/EDTA, Sigma-Aldrich St. Louis, MO, USA). The volume was adjusted for the vessel size (e.g., 1.0 mL of 0.1% trypsin/EDTA for T75 flasks), and the cultures were incubated at 37 °C. After 2–3 min, fibroblasts detached from the flask surface, as keratinocytes and fibroblasts exhibit different attachment properties, allowing for their separation.

Primary human sarcoma osteogenic cells (SaOS-2) (ATCC-HTB-85) were obtained from the American Type Culture Collection (ATCC, Manassas, VA, USA) and cultured in DMEM (Dulbecco’s Modified Eagle Medium) enriched with L-glutamine (Sigma-Aldrich, St. Louis, MO, USA) and 10% FBS and 1% antibiotics (penicillin/streptomycin). Growth occurred as mentioned above.

### 2.8. Cytotoxicity Assay

Shell extract cytotoxicity was determined on human primary fibroblasts, chondrocytes, and SaOS-2 cells. Cells were cultured with the supplementation of different concentrations (range: 31.5–500 µg/mL) of shell extracts. Cell viability was evaluated at sub-confluence in 96-well plates (Thermo Fisher Scientific, Seoul, Republic of Korea) by the MTT (3-(4,5-dimethylthiazol-2-yl)-2,5-diphenyl tetrazolium bromide) method, as previously described [34].

### 2.9. Protective Effect Under Oxidative Stress Conditions

Shell extracts were evaluated for their potential to prevent oxidative stress in 0.2 mM H_2_O_2_-treated cells. After cells reached total confluence in 96-well plates, previously filtered (0.2 μm) shell extracts (range: 3.125–100 µg/mL) were added for 24 h. Subsequently, 0.2 mM H_2_O_2_ (Sigma-Aldrich, St. Louis, MO, USA) was added, and incubation occurred for 2 h. The protective effect of shell extracts was evaluated by the MTT assay as SaOS-2 cell viability compared with untreated control.

### 2.10. Statistical Analysis

Data are presented as the means ± standard deviation (SD) of at least three independent experiments performed in triplicate. The statistical analysis was performed using Student’s *t*-test and one-way ANOVA, and differences were considered significant at *p* < 0.05 and *p* < 0.01.

### 2.11. Structural Optimization and Resources

To further investigate the antioxidant activity observed in the *C. sativa* extract, in silico studies were conducted to identify the potential biological target for ellagic and gallic acids, the extract’s two primary compounds.

First, “PASS protein prediction” was used to retrieve the list of potential targets, sorted by confidence [35]. For both ellagic and gallic acid, the target with the highest confidence was selected, and its involvement with oxidative stress was checked from the DrugBank “target section” (typing in the words “oxidative stress”) [36], which consists of 212 targets associated with cellular oxidative stress, either directly or indirectly.

The 3D structures and FASTA sequences of the validated targets were generated with AlphaFold and retrieved from the UniProt database [37], respectively.

The potential missing side chains and steric clashes in the 3D structures reported in the PDB files were addressed and resolved through molecular modeling using MODELLER v.9.3, implemented in PyMOD3.0 (PyMOL2.5 plugin) [38]. The 3D structures were analyzed and validated with PROCHECK v.3.5.4 [39,40]. To minimize high-energy intramolecular interactions before docking simulations, GROMACS 2019.3 [41] with the CHARMM36 force field [42] was employed. All parameters for the biological targets and ligands were assigned using CHARMM-GUI v.3.8 [43].

In detail, prior to conducting further simulations, the starting conformation sequence was aligned with its primary structure, allowing for the addition of any missing side chains. Loop modeling in MODELLER v.9.3 was then used to optimize the initial orientation of each loop. The structures were subsequently validated using PROCHECK, where a Ramachandran plot (assessing the backbone ϕ and ψ angles) and Chi1–Chi2 plots for side chains confirmed the validity of the starting conformations. Energy minimization was then performed on each structure using GROMACS 2019.3 with the CHARMM36 force field, aiming to eliminate steric clashes and optimize energy values.

The optimized structures were then placed in a cubic box filled with TIP3P water molecules, and the system was neutralized by adding counterions. Simulations were conducted under periodic boundary conditions. Energy minimization was carried out for 5000 steps using the steepest-descent algorithm, reaching a minimum energy with forces less than 10 kJ/mol/nm. A short 25 ns classic molecular dynamics (cMD) simulation was performed to further relax the system. During the cMD simulations, each time step was integrated with a 2 fs step size. The temperature was maintained at 300 K using a Nose–Hoover thermostat, while the system pressure was regulated at 1 atm with a Parrinello–Rahman barostat, using a damping coefficient of 1 ps−1. Bond lengths involving hydrogen atoms were constrained with the LINCS algorithm [41,44].

### 2.12. Docking Simulations and Interaction Network

Docking simulations were then performed to further unravel the interactions between ellagic and gallic acid with their respective targets as described in our previous works [5,7]. For greater consistency in docking results, the exhaustiveness was adjusted from 8 to 32 and only binding poses with an RMSD within 2 Å of the optimal docked pose were accepted; all other parameters were kept as default. The 3D structures of gallic acid and ellagic acid were obtained from the PubChem database [44,45], with compound IDs 370 and 5281855, respectively, and downloaded in SDF format. A box was configured around the residues previously shown to be involved in interactions with their inhibitors using DockingPie2.0 [46] integrated into PyMOL 3.0, with AutoDock Tools v.4.2 [36,47].

The AutoDock/VinaXB v.1.1.2 tool performed docking simulations [48]. Protein and ligand files were converted using MGLTOOLS v.1.5.7 scripts, while Gasteiger partial charges were added with OpenBabel v.3.1.0 [49]. The 3D structures of gallic acid and ellagic acid were obtained from the PubChem database (CID: 370 and 5281855, respectively) [44].

The interaction networks were analyzed using the P.L.I.P. v. 2.3.0 tool [50], and sequence alignments to identify key target residues were conducted with ClustalW v.2.1 [51,52].

## 3. Results

### 3.1. C. sativa Extracts’ Total Phenolic Contents

The extraction yields for the three chestnut cultivars and their total phenolic contents were evaluated and shown in Table 1. The total phenolic contents of the cultivars varied from 7.041 (RC) to 1.893 (RM) GAE/100 g fresh material (GAE, gallic acid equivalent). The cultivar *Cecio* (RC) showed the highest total phenolic content of 7 GAE/100 g fresh material, followed by the cultivar *Bastarda Rossa* (RB) with 4.8 GAE/100 g fresh material of total phenolics. The lowest phenolic content was reported for the cultivar *Marroni* (RM), with 1.8 GAE/100 g fresh material.

These values confirmed that the mixture EtOH:H_2_O at 70:30 is optimal for the extraction of phenolic compounds, indicating the potential of *Cecio* and *Bastarda Rossa* chestnut shell extracts to formulate nutraceuticals or cosmetics as natural antioxidants.

### 3.2. C. sativa Extracts’ Flavonoid Contents

The flavonoid content of the extracts of the three cultivars was evaluated and reported in Table 2. The total flavonoid content varied from 319.2 (RM) to 1000 (RC) quercetin/100 g fresh material.

*Cecio* (RC) showed the highest value, followed by the cultivar *Bastarda Rossa* (RB) and then *Marroni* (RM). These findings would suggest that the total flavonoid content in the three cultivars follows the line of TPC and contributes to the antioxidant power of the extracts.

### 3.3. C. sativa Extracts’ Antioxidant Activity

The total antioxidant activities of three different chestnut cultivar shell extracts were evaluated by the FRAP assay. As shown in Table 3, the highest total antioxidant activity was found in cultivar *Cecio* (RC) with the value of 146.5 AAE/100 g fresh material, followed by *Bastarda Rossa* (RB) and *Marroni* (RM), with the values of 91.5 and 34.9 AAE/100 g fresh material, respectively. These data align with previous findings that *Cecio* and *Bastarda Rossa* are the best cultivars for their antioxidant power.

### 3.4. C. sativa Extracts’ Antiradical Activity

**Figure 1:**
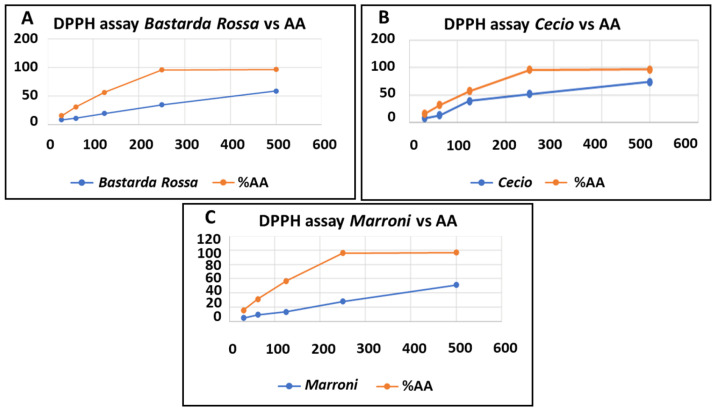
DPPH assay. Absorbance decrease in three cultivar extracts analyzed compared to control, ascorbic acid (AA). Antioxidant activity (expressed as %AA) of chestnut varieties was measured at varying extract concentrations (0–500 µg/mL). (**A**–**C**) represent cultivars *Bastarda Rossa*, *Cecio*, and *Marroni*, respectively.

The DPPH colorimetric assay is based on the ability of reducing molecules to scavenge the 2,2-diphenyl-1-picrylhydrazyl synthetic radical dissolved in an alcoholic solution, thus obtaining its neutral form. The process is monitored following the conversion of the violet-colored radical into a pale-yellow solution at 517 nm, and the antiradical activity is reported at a percentage decrease in such absorbance. The antiradical activity was evaluated by the DPPH assay and the results are shown in Figure 1 and Table 4. Even in this case, the cultivar *Cecio* was the most effective in terms of antiradical activity with a 76.6% Abs decrease.

Combining the results obtained from the DPPH colorimetric assay and FRAP assay, information was gathered about the redox properties and radical-scavenging activity induced by electron and hydrogen transfer, which are key processes related to tannins’ antioxidant capacity.

The antioxidant activity of the three cultivar extracts and their TPC values were positively correlated (antioxidant capacity vs. TPC: *p* = 0.009159382; antiradical power vs. TPC: *p* = 0.012930563), implying that the measured antioxidant capacity is reasonably attributable to the phenolic compounds present in the extracts. Additionally, *Cecio* and *Bastarda Rossa* cultivars represented better sources of bioactive antioxidant molecules than the *Marroni* cultivar.

### 3.5. Phenolic Compound Determination by NMR Analysis

Bioactive compounds of *Bastarda Rossa*, *Cecio*, and *Marroni* shells were elucidated by the NMR metabolomics approach. Indeed, the metabolite profiling associated with the antioxidant potential of chestnut shell extracts of the three main cultivars of *Monte Amiata* PGI represents an important step in the bioactive compound′s characterization due to the chestnut biodiversity in this region.

**Figure 2:**
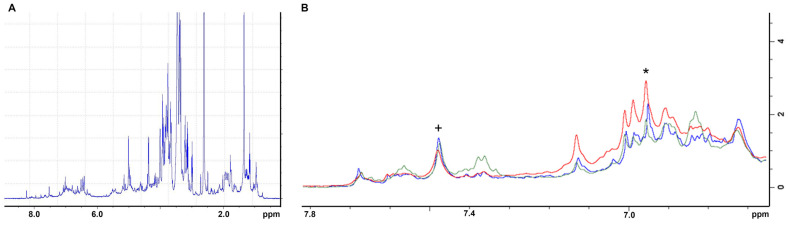
(**A**) The 1H NMR spectrum of an aqueous extract. (**B**) The aromatic region of the spectrum shows signals of gallic acid (*) and ellagic acid (+) for the cultivar *Bastarda Rossa* (RB, blue), *Cecio* (RC, red), and *Marroni* (RM, green). Signal integration allowed for polyphenol quantification, reported in Table 5.

Through the analysis of 1H NMR spectra, gallic and ellagic acid were identified as the main components in the aqueous extracts of the shells from the three cultivars (see Figure 2). As shown in Table 5, NMR quantification indicates that *Bastarda Rossa* (RB) had the highest concentration of ellagic acid, followed by *Cecio* (RC) and *Marroni* (RM). In contrast, RC had a greater gallic acid concentration than both RB and RM. Overall, *Marroni* was found to have the lowest levels of these compounds.

**Figure 3:**
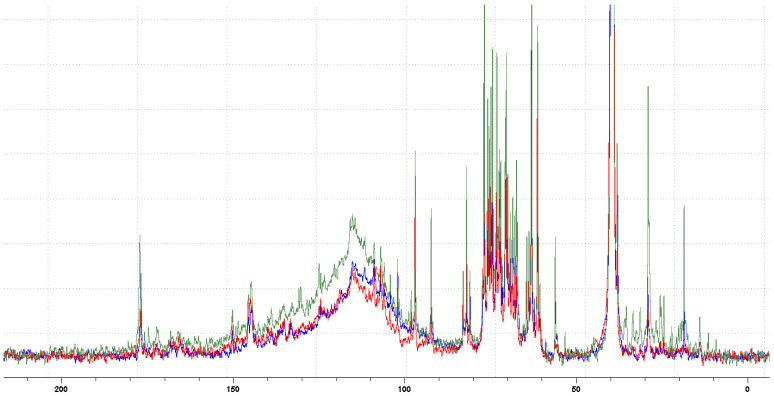
13C NMR normalized spectra of *Bastarda Rossa* (RB, blue), *Cecio* (RC, red), and *Marroni* (RM, green).

The screening conducted using 13C NMR spectroscopy provided a quantitative profile of the phytochemicals present in each material, as shown in Figure 3 and Table 6. This study demonstrated that 1D 13C data improve the identification of metabolites and facilitate the better separation of groups for comparison. The 13C NMR spectrum of the hydroalcoholic shell extracts from the three cultivars revealed signals in the 140–160 ppm region, indicating the presence of carbons derived from phenolic compounds. Additionally, alkoxy carbons were identified in the region from 50 to 110 ppm, likely associated with sugars such as glucose. In the *Marroni* (RM) cultivar, these sugars appear to be more abundant than in the other cultivars (refer to Table 6). This could be attributed to the fact that *Marroni* is a cultivated variety selected for specific nut qualities and consumer uses, such as candying, roasting, boiling, and drying, which require a sweeter flavor. Furthermore, the *Marroni* tree has more specific growing requirements and is generally less productive in terms of the number of chestnuts produced per tree compared to ordinary chestnuts. The findings regarding phenolic compounds are consistent with previous data and indicate that the *Cecio* (RC) cultivar is the richest in phenolic content.

### 3.6. Citotoxicity of C. sativa Shell Extracts

The cytotoxic effect of the three extracts was assessed in the SaOS-2 cell line, primary chondrocytes, and human primary fibroblasts.

**Figure 4:**
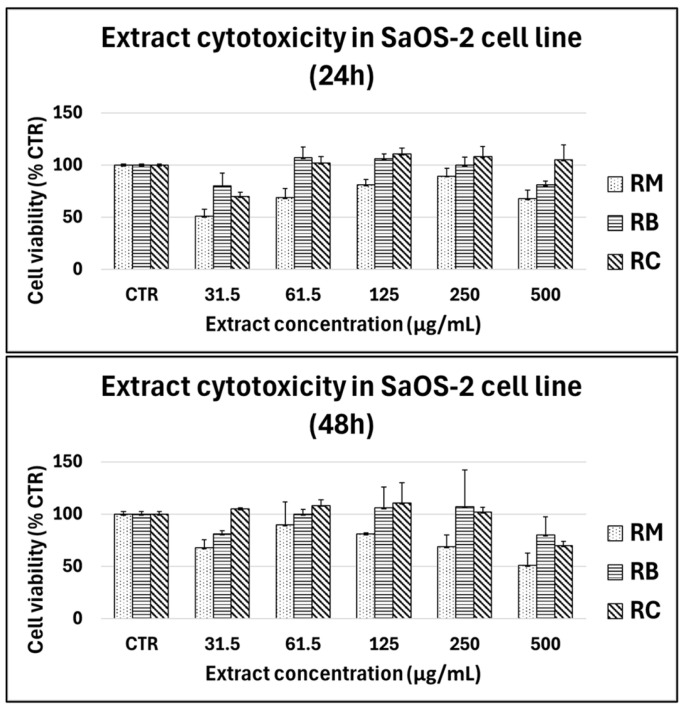
Cytotoxicity of chestnut extracts (RM: *Marroni*, RB: *Bastarda Rossa*, RC: *Cecio*) on SaOS-2 cells at varying concentrations (31.5–500 µg/mL) after 24 h (**top panel**) and 48 h (**bottom panel**) of treatment. Cell viability was measured using the MTT assay, and the results are expressed as a percentage of the untreated control group (CTR). Extract concentrations ranged from 31.5 to 500 µg/mL, with bars representing the mean ± standard deviation from three independent experiments.

Cells were treated with increasing concentrations of the three different extracts (31.5–500 µg/mL) for 24 h and 48 h and the cytotoxic effect on cell viability was assessed by the MTT assay (Figure 4).

Results showed that for all cultivars, no cytotoxic effect was observed in SaOS-2 or primary chondrocytes and human primary fibroblasts for concentrations ranging from 31.5 to 250 µg/mL, whilst extracts reduced the viability at 500 µg/mL. A cytotoxic effect on the viability of SaOS-2 cells was observed at 24 h at 500 µg/mL only for the cultivar *Marroni*, but the extract did not affect the viability until the concentration of 250 µg/mL also at 48 h.

**Figure 5:**
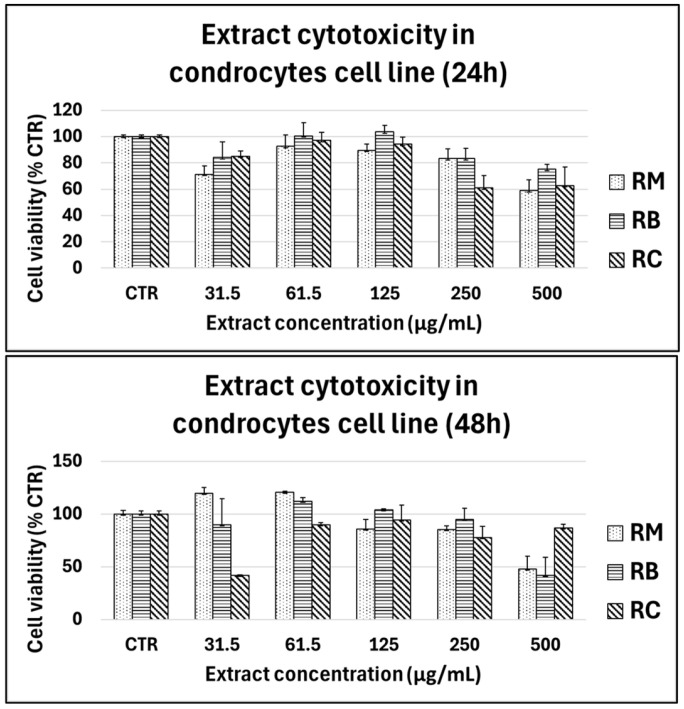
Cytotoxicity of chestnut extracts (RM: *Marroni*, RB: *Bastarda Rossa*, RC: *Cecio*) on chondrocyte varying concentrations (31.5–500 µg/mL) after 24 h (**top panel**) and 48 h (**bottom panel**) of treatment. Cell viability was measured using the MTT assay, and the results are expressed as a percentage of the untreated control group (CTR). Extract concentrations ranged from 31.5 to 500 µg/mL, with bars representing the mean ± standard deviation from three independent experiments.

The three extracts were safe even on primary human chondrocytes, in concentrations ranging from 31.5 to 250 µg/mL (Figure 5).

**Figure 6:**
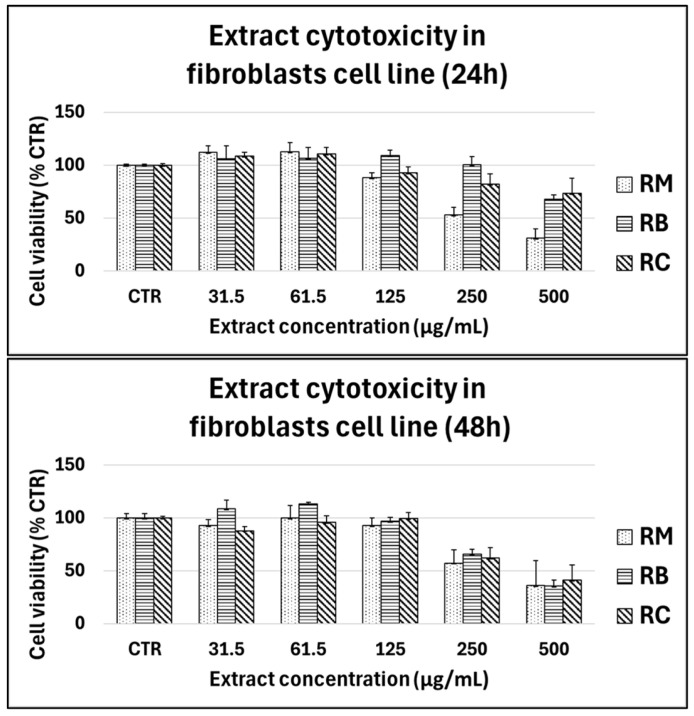
Cytotoxicity of chestnut extracts (RM: *Marroni*, RB: *Bastarda Rossa*, RC: *Cecio*) on fibroblasts at varying concentrations (31.5–500 µg/mL) after 24 h (**top panel**) and 48 h (**bottom panel**) of treatment. Cell viability was measured using the MTT assay, and the results are expressed as a percentage of the untreated control group (CTR). Extract concentrations ranged from 31.5 to 500 µg/mL, with bars representing the mean ± standard deviation from three independent experiments.

In human primary fibroblasts (Figure 6), the three extracts showed a similar trend, being the three cultivars cytotoxic at the concentrations 250–500 µg/mL, but safe at minor concentrations. According to the obtained results, the extract did not decrease the cell viability in the range of 31.5–250 μg/mL in both cell types, supporting their potential safety in human cells at appropriate concentrations.

### 3.7. Evaluation of Antioxidant Power of Three Cultivar Extracts

Strong evidence states that oxidative stress is responsible for the pathophysiology of the aging process and may also be involved in the pathogenesis of atherosclerosis, neurodegenerative diseases, cancer, and diabetes. Epidemiological evidence indicated a link between dietary intake of antioxidants and bone health and metabolism, while numerous studies showed the health-promoting properties of polyphenols, providing additional mechanisms through which they promote skeletal health by reducing resorption caused by high oxidative stress [8,53].

**Figure 7:**
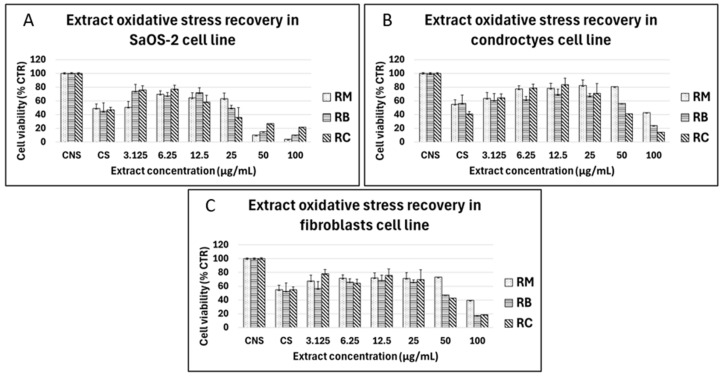
Oxidative stress recovery in various cell lines treated with chestnut extracts. RM = *Marroni*; RB = *Bastarda Rossa*; RC = *Cecio*. (**A**) SaOS-2; (**B**) chondrocytes; and (**C**) fibroblasts, at different concentrations (3.125–100 µg/mL). Oxidative stress was induced in cells using hydrogen peroxide (H_2_O_2_), with untreated stressed cells (CNS) and untreated non-stressed cells (CS) serving as controls. Cell viability was measured using MTT, and results are expressed as a percentage of the untreated non-stressed control (CS). Data represent the mean ± standard deviation from three independent experiments.

In this study, chestnut shells were used to obtain bioactive compounds given their richness in phenolic compounds with antioxidant power, responsible for biological effects that contribute to benefits in human health and potential food applications in the development of functional foods. The three cultivar extracts were also assessed for their ability to counteract the ROS effects on cultured human cells, thereby legitimizing their use in the alimentary field. Briefly, SaOS-2 cells, human primary chondrocytes, and human primary fibroblasts were exposed to H_2_O_2_ (0.2 mM), which, in preliminary experiments, reduced cell viability by 50% (Figure 7).

For SaOS-2 cells, all three extracts showed a viability increase, compared to treated controls, in the range of 3125 to 25 μg/mL, reaching a recovery of over 50% with *Bastarda Rossa* (RB) and *Cecio* (RC). Results on primary chondrocytes were particularly interesting, with a viability recovery over 50% for RC at the concentration of 2.5 μg/mL. The same recovery was found for RM in the range of 6.25–50 μg/mL. RB was a little less efficient, but, at 12.5 μg/mL, it allowed for recovery of approximately 25% of the viability compared to the stressed control. On human primary fibroblasts, all the tested extracts showed a noticeable ability to recover from oxidative stress. The RC extract was the best for antioxidant ability on cells, showing a higher percentage of viability recovery (Figure 7). Overall, for every extract, a recovery of cell viability was assessed from 30 to 50% for RC on chondrocytes and SaOS-2, and 25% for human primary fibroblasts. In total, 6.25 and 12.5–25 μg/mL are the ranges of better concentrations presenting the highest protective effect in all the tested extracts in the SaOS-2 cell line, and in chondrocytes and fibroblasts, respectively (Figure 7).

These results further contributed to evaluating the high potential of this chestnut byproduct as a new source of antioxidant compounds to be used in various fields, such as developing functional foods and nutraceuticals.

### 3.8. In Silico Results

#### Target Identification and Docking Simulations

In silico studies were meant to confirm the in vitro-observed antioxidant activity by predicting the biological targets for the two most abundant compounds within the extracts (ellagic acid and gallic acid) and evaluating their interactions at the molecular level.

To identify potential targets of the two most abundant compounds within the extract, namely ellagic and gallic acid, a ligand-based virtual screening was conducted through the “PASS Protein Prediction” [35], which, for each compound, sorted the predicted targets by confidence. The most confident target was then checked for its presence in the complete oxidative stress target network, identified using the DrugBank database “target section” [36], which consists of 212 targets associated with cellular oxidative stress, either directly or indirectly. The individuated targets were serine/threonine-protein kinase NEK6 for ellagic acid and carbonic anhydrase III for gallic acid, respectively, displaying 77% and 87% confidence. For each target, its primary sequence was retrieved from the UniProt database (serine/threonine-protein kinase NEK6, UniprotKB entry: Q9HC98; carbonic anhydrase III, UniprotKB entry: P07451). AlphaFold generated the targets’ 3D structure, further optimized through molecular modeling to solve structure gaps and steric hindrances.

**Figure 8:**
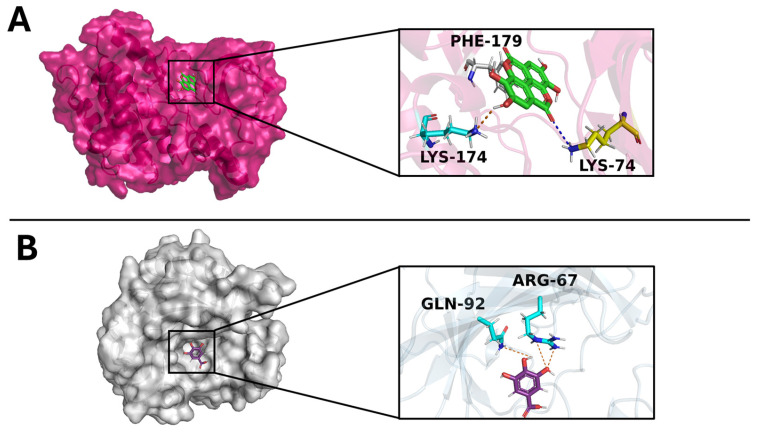
An overview of the target/ligand complexes. (**A**) is ellagic acid in complex with the NEK6 kinase 3D structure; (**B**) is gallic acid in complex with the carbonic anhydrase III 3D structure. The enlarged pictures display the interaction network established among the target-binding residues and the ligand. The binding residues involved in hydrogen bonds, salt bridges, and hydrophobic interactions are labeled cyan, yellow, and gray, respectively. Hydrogen bonds and salt bridges are pictured as orange and blue dashed lines, respectively.

Gallic acid and ellagic acid were then evaluated against their targets using docking simulations (Figure 8). Two strategies were employed to select the top complexes: (i) selecting compounds with a binding free energy (docking score) lower than −6 kcal/mol and (ii) assessing the compound’s ability to form strong interactions with consensus binding residues of the target. The results indicated a docking score of −6.9 kcal/mol for the complex ellagic acid/NEK6, and a docking score of −5.7 kcal/mol for the complex gallic acid/carbonic anhydrase III.

The potential inhibitory effects of ellagic and gallic acid on their targets were further explored by analyzing the interaction networks, showing wide polar and non-polar interactions between each compound and its target that were identified. Specifically, ellagic acid formed a broad interaction network with key NEK6 residues, such as Phe-179 (hydrophobic interaction), Lys-174 (hydrogen bond), and Lys-74 (salt bridge) [54]. Likewise, gallic acid established hydrophobic interactions with carbonic anhydrase III critical residues, that is, Arg-67 and Gln-92 [55].

Our findings suggest that these compounds exhibit binding capabilities with their targets in key residues, which may result in the potential inhibitory activity displayed in vitro. Overall, computational studies confirmed the in vitro results and provided additional insights on the mechanism of action of the extracts’ major compounds, through which they exert their activity.

## 4. Discussion

Natural sources like plants have long presented promising alternatives for the development of bioproducts with healthy properties. This is a field of growing importance, especially within the food and nutraceutical industries, where demand for natural, sustainable ingredients has risen. For centuries, natural compounds derived from plants have been foundational in traditional medicine systems, including traditional Chinese medicine. These compounds offer therapeutic effects by influencing crucial biological processes such as cell proliferation, angiogenesis, survival, and apoptosis. Additionally, plant-derived bioactive molecules are valued for their strong antioxidant and anti-inflammatory properties, which make them integral to therapeutic practices aimed at combating oxidative stress and chronic inflammation [56]. This knowledge of plant bioactive molecules laid the foundation for a transition toward the use of sustainably derived natural compounds to develop health-focused products. This shift is particularly evident in the food and nutraceutical sectors, where sourcing effective bioactive molecules from natural, plant-based materials became a priority. As consumers are becoming more healthy and environmentally aware, there is an increasing demand for sustainable products. This growing interest might open new routes for innovation, especially in the creation of functional foods and natural health supplements that promote wellness through diets [57,58,59].

One of the most reliable sources of such bioactive compounds is chestnut (*Castanea sativa*), a tree species whose cultivation is deeply rooted in Italian agricultural traditions. However, in recent years, chestnut cultivation has lowered, particularly due to the diminishing economic viability of traditional chestnut-based products, and, while the decline in chestnut cultivation poses challenges, adopting a holistic approach that combines economic, environmental, and technological strategies can ensure sustainability. By leveraging chestnut byproducts and promoting eco-friendly practices, it is possible to sustainably obtain antioxidant-rich extracts while supporting the revival of this valuable crop [60]. This decline urged the need to explore new applications for chestnut byproducts, which account for approximately 10–15% of the total chestnut weight. These byproducts, often discarded as waste, in fact contain a wealth of valuable bioactive compounds that could contribute significantly to food sustainability and nutraceutical innovation [61,62]. This approach would also reduce pollution and CO_2_ emissions from burning the chestnut burrs to ensure optimal tree growth [17].

This study investigates the underexplored potential of chestnut shells as a rich source of bioactive compounds like tannins, phenolic acids, and flavonoids, which are known for their antioxidant, anti-inflammatory, and antimicrobial properties. The two most abundant compounds found by NMR from this study were ellagic acid and gallic acid, both presenting a sustainable alternative to synthetic antioxidants in food systems. Specifically, chestnut shell-derived antioxidants can be incorporated into food products to combat oxidative damage, a contributing factor to chronic diseases such as cancer, cardiovascular disease, and neurodegenerative disorders [5,7,63]. Additionally, chestnut extracts could be used in the nutraceutical industry for supplements targeting oxidative stress and inflammation [19,20]. Beyond health, their antimicrobial properties make them valuable for developing functional foods that promote gut health and food preservation, potentially replacing synthetic preservatives with natural alternatives by combating both food-borne and health-threatening pathogens [7,21]. Furthermore, these bioactive compounds could be harnessed in skincare products, offering natural protection from oxidative stress due to environmental pollutants and UV radiation [22,23].

Importantly, the use of chestnut burrs aligns with the principles of the circular bioeconomy by reducing food waste while creating high-value products [64].

As part of this study, the hydroalcoholic extracts from three *Monte Amiata* PGI chestnut cultivars—*Bastarda Rossa* (RB), *Cecio* (RC), and *Marroni* (RM)—were biochemically characterized for phenolic, flavonoid, and antioxidant content.

The FRAP and DPPH assays were selected due to their ease of use and proven reliability for assessing antioxidant activity. These methods are widely recognized for their straightforward protocols, adaptability to a high-throughput analysis, and consistent reproducibility. They effectively provide foundational antioxidant profiles, enabling the quick evaluation of the potential of various plant-based compounds. Furthermore, their robust nature makes them suitable for initial screening and comparative studies, offering valuable insights into the efficacy and bioactivity of the extracts under investigation [65,66,67]. These assays provide a straightforward method to compare the radical-scavenging capacities of various extracts, and they are particularly useful for establishing preliminary antioxidant profiles, guiding the selection of extracts for more comprehensive studies.

RC showed the highest concentration of bioactive compounds such as gallic acid, ellagic acid, and flavonoids, and consequently, its potential application in the nutraceutical field. RM, with a milder profile of these molecules due to selective breeding, appeared more suited for food consumption, aligning well with evolving consumer behavior toward functional foods [22,23,24]. Then, the three cultivars’ extracts were assessed for cytotoxicity and protection from oxidative stress in three different cell lines: SaOS-2, fibroblasts, and chondrocytes. Although a slight variation in the cell model viability response used in this study was noted upon treatment with the three cultivar extracts, none of the considered extracts yielded a toxic effect in the cultured cells in a wide range of concentrations. The variations in cellular responses to the extracts could be attributed to the distinct biological characteristics of each cell type. SaOS-2 cells, an osteosarcoma cell line, are typically more sensitive to oxidative stress and reactive oxygen species (ROS) due to their heightened metabolic activity. As shown in several studies, osteosarcoma cells can exhibit significant reductions in viability when exposed to high concentrations of plant extracts that induce oxidative damage [68]. Fibroblasts, on the other hand, tend to be more resilient to oxidative stress. They have evolved efficient antioxidant defense mechanisms, allowing them to better tolerate prolonged exposure to potentially harmful compounds. Studies have demonstrated that fibroblasts can endure higher concentrations of bioactive compounds without a drastic decrease in cell viability [69]. Chondrocytes exhibit slower metabolic activity and lower antioxidant capacities, making them more vulnerable to oxidative stress. This characteristic makes chondrocytes more sensitive to prolonged exposure to high concentrations of plant extracts, as evidenced by similar findings in other studies on cartilage cells [70]. These inherent differences in cellular resilience to oxidative stress and metabolic activity explain the observed differential responses in cell viability across the different cell types used in this study upon treatment with the chestnut extract. In regards to the cytoprotective activity, the *Cecio* and *Bastarda Rossa* extracts demonstrated strong cytoprotective effects against H_2_O_2_-induced oxidative stress in all cell lines, likely due to their elevated levels of gallic acid, ellagic acid, and flavonoids, which may modulate oxidative stress pathways or stimulate cellular repair mechanisms (as shown by cell viability percentages compared to the untreated controls). According to the literature body, all these bioactive compounds display well-documented protective activities against oxidative stress [19], underlining the prospects of chestnut byproducts to support food waste, adding value through innovative applications in food sustainability and nutraceuticals.

To elucidate more the possible mechanism of action of the extracts’ bioactive compounds, in silico studies were conducted. The potential targets for ellagic acid and gallic acid, the two most abundant compounds within the extracts, were pinpointed as the serine/threonine-protein kinase NEK6 and carbonic anhydrase III. The following docking simulation provided useful insights into the potential interaction between each ligand with its target. As a result, ellagic acid and gallic acid both exhibited a high binding free energy score, triggering hydrogen bonds, salt bridges, and hydrophobic interactions with key target residues [54,55]. Altogether, these simulations strongly strengthened the experimental evidence provided in our study and demonstrated the potential ability of the extracts’ bioactive compounds to interfere with the target biological function by interacting with critical target residues.

This study found that the three analyzed chestnut shell extracts from *Cecio*, *Bastarda Rossa*, and *Marroni* cultivars could significantly contribute to food system innovation as naturally innovative sources of antioxidant compounds.

Given the promising bioactivity of the chestnut burr extracts, particularly the presence of phenolic compounds such as ellagic acid and gallic acid, there is potential for translating these findings into practical applications within the food and nutraceutical industries [8,9,10,11,12,13]. These compounds are well known for their antioxidant and antimicrobial properties, making them ideal candidates for incorporation into functional foods, natural preservatives, or supplements. For instance, ellagic acid demonstrated potential in reducing oxidative stress and inflammation, which could be leveraged in formulating health supplements aimed at supporting cardiovascular or anti-cancer benefits [19,20,22,23]. Additionally, the antimicrobial properties observed in these extracts could lead to their application as natural preservatives in food products, offering an eco-friendly alternative to synthetic preservatives [7,21].

Harnessing these byproducts from chestnut processing not only supports circular bioeconomic goals but also aligns with global efforts to enhance food sustainability, reduce food loss, and potentially improve food security by creating value-added health-promoting solutions from otherwise discarded resources.

However, while we value the importance of investigating the stability, bioavailability, dosages, and practical applications of the three extracts within food systems, this study primarily focuses on the antioxidant potential of chestnut burr extracts at the in vitro level. These aspects might be explored in future studies, as they represent an essential next step in understanding how such bioactive compounds can be incorporated into food or nutraceutical formulations. This work serves as a foundation for future research aimed at harnessing the benefits of chestnut-derived compounds in sustainable food production and health-related products [19].

## 5. Conclusions

Our study was conducted alongside a circular bioeconomy framework, aiming to repurpose chestnut shells for their valuable biological properties. By focusing on three cultivars of *Castanea sativa* shells—*Cecio*, *Bastarda Rossa*, and *Marroni*—the antioxidant potential and phenolic content of their hydroalcoholic extracts were investigated. Findings from this study demonstrated that the shell extracts from the *Cecio* and *Bastarda Rossa* cultivars showed notably higher antioxidant activity and concentrations of key bioactive compounds, including ellagic acid, gallic acid, and flavonoids. Fundamentally, ellagic and gallic acid were the two most abundant metabolites to be found. The biochemical characterization further confirmed that *Cecio* and *Bastarda Rossa* are particularly promising cultivars as their extracts exhibit strong cytoprotective and antioxidant effects in cellular models under oxidative stress. H_2_O_2_-induced cytotoxicity and oxidative damage were significantly reduced in human cells, underscoring the protective potential of their phenolic compounds against aging-related oxidative stress, representing valuable sources of bioactive compounds with potential applications in nutraceuticals, food innovation, and food sustainability.

To further investigate the potential antioxidant activity of the cultivars’ extracts, in silico studies were performed to identify the target for the two majorly represented compounds in the extracts, namely ellagic acid and gallic acid. Their interaction with the target was explored through docking simulations and an interaction network analysis, displaying a range of interactions, which would potentially justify the inhibitory effect of the ligands on their respective targets.

Ellagic and gallic acid were selected as the most abundant compounds in the extracts. In silico findings indicated their potential bioactivity against their predicted targets, warranting further investigation. Importantly, this study was not focused on proposing potential target inhibitors, rather to highlight the importance and the innovation to align the circular bioeconomy principles by identifying natural wastes as antioxidant sources, reducing time, and costs, and minimizing environmental pollutants. Exploring the extracts’ potential within a sustainable, integrated bioprocessing approach offers an eco-friendly pathway to assess the biological activity of plant byproduct compounds. However, further investigations are needed to explore their stability, bioavailability, dosages, and potential uses in nutraceuticals or functional foods.

## Figures and Tables

**Table 1:** Total Phenolic Content of the three analyzed cultivars: *Bastarda Rossa* (RB); *Cecio* (RC); *Marroni* (RM).

Samples	Extraction Yield (%)	GAE/mg of Extract	GAE/100 g of Fresh Material
RB	16.64	0.292961609	4.866
RC	20.84	0.338665448	7.041
RM	7.6	0.257312614	1.893

**Table 2:** Flavonoid Content of the three analyzed cultivars: *Bastarda Rossa* (RB); *Cecio* (RC); *Marroni* (RM).

Samples	Extraction Yield (%)	QE/mg of Extract	QE/100 g of Fresh Material
RB	16.64	0.0483	798.7
RC	20.84	0.0508	1000
RM	7.6	0.0425	319.2

**Table 3:** Antioxidant activity of three cultivars analyzed by FRAP assay: *Bastarda Rossa* (RB); *Cecio* (RC); *Marroni* (RM).

Samples	Extraction Yield (%)	mmol AAE/mg Extract	AAE/100 g of Fresh Material
RB	16.64	5.530995	91.520
RC	20.84	7.037723	146.5
RM	7.6	4.564747	34.96

**Table 4:** Antiradical activity of three cultivar extracts analyzed by DPPH assay. Results are expressed as Abs decrease (%) at 517 nm. *Bastarda Rossa* (RB); *Cecio* (RC); *Marroni* (RM).

Samples	Extraction Yield (%)	% Abs Decrease, 517 nm
RB	16.64	61.11
RC	20.84	76.6
RM	7.6	52.8

**Table 5:** The concentration of (+) ellagic and (*) gallic acid reported as mg/100 mg extract in the three cultivars determined by 1H NMR spectroscopy. *Bastarda Rossa* (RB); *Cecio* (RC); *Marroni* (RM).

Sample	RB	RC	RM
(+) ellagic acid	0.61 ± 0.12	0.56 ± 0.11	0.49 ± 0.10
(*) gallic acid	0.41 ± 0.08	0.56 ± 0.11	0.22 ± 0.04

**Table 6:** Phytochemicals present in the three cultivars obtained by 13C NMR spectroscopy. (Quantities were normalized to 100 in relation to the internal calibrant peak.) *Bastarda Rossa* (RB); *Cecio* (RC); *Marroni* (RM).

	C_alkyl_ (0–35 ppm)	C_alkoxy_ (50–100 ppm)	C_aromatic_ (100–140 ppm)	C_phenolic_ (140–160 ppm)	C_carboxyl/carbonyl_ (160–215 ppm)
RB	2.5	47.0	63.2	5.5	4.1
RC	3.4	38.5	54.13	7.9	1.9
RM	14.5	60.7	81.2	7.0	6.3

## Data Availability

The original contributions presented in the study are included in the article; further inquiries can be directed to the corresponding author.

## References

[1] Chang X., Liu F., Lin Z., Qiu J., Peng C., Lu Y., Guo X. (2020). Phytochemical Profiles and Cellular Antioxidant Activities in Chestnut (*Castanea mollissima* BL.) Kernels of Five Different Cultivars. Molecules.

[2] Cravotto C., Grillo G., Binello A., Gallina L., Olivares-Vicente M., Herranz-López M., Micol V., Barrajón-Catalán E., Cravotto G. (2022). Bioactive Antioxidant Compounds from Chestnut Peels through Semi-Industrial Subcritical Water Extraction. Antioxidants.

[3] Echegaray N., Gómez B., Barba F.J., Franco D., Estévez M., Carballo J., Marszałek K., Lorenzo J.M. (2018). Chestnuts and By-products as Source of Natural Antioxidants in Meat and Meat Products: A Review. Trends Food Sci. Technol..

[4] Comandini P., Lerma-García M.J., Simó-Alfonso E.F., Toschi T.G. (2014). Tannin Analysis of Chestnut Bark Samples (*Castanea sativa* Mill.) by HPLC-DAD-MS. Food Chem..

[5] Trezza A., Geminiani M., Cutrera G., Dreassi E., Frusciante L., Lamponi S., Spiga O., Santucci A. (2024). A Drug Discovery Approach to Reveal a Novel Antioxidant Natural Source: The Case of Chestnut Burr Biomass. Int. J. Mol. Sci..

[6] De Vasconcelos M.C., Bennett R.N., Rosa E.A., Ferreira-Cardoso J.V. (2010). Composition of European Chestnut (*Castanea sativa* Mill.) and Association with Health Effects: Fresh and Processed Products. J. Sci. Food Agric..

[7] Trezza A., Barletta R., Geminiani M., Frusciante L., Olmastroni T., Sannio F., Docquier J.-D., Santucci A. (2024). Chestnut Burrs as Natural Source of Antimicrobial Bioactive Compounds: A Valorization of Agri-Food Waste. Appl. Sci..

[8] Sorice A., Siano F., Capone F., Guerriero E., Picariello G., Budillon A., Ciliberto G., Paolucci M., Costantini S., Volpe M.G. (2016). Potential Anticancer Effects of Polyphenols from Chestnut Shell Extracts: Modulation of Cell Growth, and Cytokinomic and Metabolomic Profiles. Molecules.

[9] Pinto D., Braga N., Silva A.M., Costa P., Delerue-Matos C., Rodrigues F. (2019). Valorization of Fruit Processing By-Products.

[10] Liberti A., Goretti G., Russo M.V. (1983). PCDD and PCDF formation in the combustion of vegetable wastes. Chemosphere.

[11] Silva V., Falco V., Dias M.I., Barros L., Silva A., Capita R., Alonso-Calleja C., Amaral J.S., Igrejas G., Ferreira C.F.R. (2020). Evaluation of the Phenolic Profile of *Castanea sativa* Mill. By-Products and Their Antioxidant and Antimicrobial Activity against Multiresistant Bacteria. Antioxidants.

[12] Neves J.M., Matos C., Moutinho C., Queiroz G., Gomes L.R. (2009). Ethnopharmacological Notes about Ancient Uses of Medicinal Plants in Trás-os-Montes (Northern Portugal). J. Ethnopharmacol..

[13] Corregidor V., Antonio A.L., Alves L.C., Cabo Verde S. (2020). Castanea sativa Shells and Fruits: Compositional Analysis by Proton Induced X-Ray Emission. Nucl. Instrum. Methods Phys. Res. Sect. B.

[14] Bongaarts J. (2009). Human Population Growth and the Demographic Transition. Philos. Trans. R. Soc. Lond. B Biol. Sci..

[15] Di Vaio A., Hasan S., Palladino R., Hassan R. (2023). The Transition Towards Circular Economy and Waste within Accounting and Accountability Models: A Systematic Literature Review and Conceptual Framework. Environ. Dev. Sustain..

[16] Venkatesh G. (2022). Circular Bio-economy—Paradigm for the Future: Systematic Review of Scientific Journal Publications from 2015 to 2021. Circ. Econ. Sustain..

[17] Ghangrekar M.M., Das S., Das S., Pandey A., Tyagi R.D., Varjani S. (2021). 15—Microbial Electrochemical Technologies for CO_2_ Sequestration. Biomass, Biofuels, Biochemicals.

[18] Faraoni P., Laschi S. (2024). Bioactive Compounds from Agrifood Byproducts: Their Use in Medicine and Biology. Int. J. Mol. Sci..

[19] Hadidi M., Liñán-Atero R., Tarahi M., Christodoulou C.M., Aghababaei F. (2024). The Potential Health Benefits of Gallic Acid: Therapeutic and Food Applications. Antioxidants.

[20] Golmei P., Kasna S., Roy K.P., Kumar S. (2024). A Review on Pharmacological Advancement of Ellagic Acid. J. Pharmacol. Pharmacother..

[21] De R., Sarkar A., Ghosh P., Ganguly M., Karmakar B.C., Saha D.R., Halder A., Chowdhury A., Mukhopadhyay A.K. (2018). Antimicrobial Activity of Ellagic Acid against *Helicobacter pylori* Isolates from India and during Infections in Mice. J. Antimicrob. Chemother..

[22] Khan B.A., Mahmood T., Menaa F., Shahzad Y., Yousaf A.M., Hussain T., Ray S.D. (2018). New Perspectives on the Efficacy of Gallic Acid in Cosmetics & Nanocosmeceuticals. Curr. Pharm. Des..

[23] Evtyugin D.D., Magina S., Evtuguin D.V. (2020). Recent Advances in the Production and Applications of Ellagic Acid and Its Derivatives: A Review. Molecules.

[24] Younesi F.S., Miller A.E., Barker T.H., Rossi F.M.V., Hinz B. (2024). Fibroblast and Myofibroblast Activation in Normal Tissue Repair and Fibrosis. Nat. Rev. Mol. Cell Biol..

[25] Goldring M.B. (2012). Chondrogenesis, Chondrocyte Differentiation, and Articular Cartilage Metabolism in Health and Osteoarthritis. Ther. Adv. Musculoskelet. Dis..

[26] Marcucci G., Domazetovic V., Nediani C., Ruzzolini J., Favre C., Brandi M.L. (2023). Oxidative Stress and Natural Antioxidants in Osteoporosis: Novel Preventive and Therapeutic Approaches. Antioxidants.

[27] Dai J., Mumper R.J. (2010). Plant Phenolics: Extraction, Analysis and Their Antioxidant and Anticancer Properties. Molecules.

[28] Liyana-Pathirana C., Shahidi F. (2005). Optimization of Extraction of Phenolic Compounds from Wheat Using Response Surface Methodology. Food Chem..

[29] Singleton V.L., Orthofer R., Lamuela-Raventós R.M. (1999). Analysis of Total Phenols and Other Oxidation Substrates and Antioxidants by Means of Folin-Ciocalteu Reagent. Methods Enzymol..

[30] Chang C.F., Yang M.-H., Wen H.-M., Chern J.C. (2020). Estimation of Total Flavonoid Content in Propolis by Two Complementary Colorimetric Methods. J. Food Drug Anal..

[31] Jayaprakasha G.K., Singh R.P., Sakariah K.K. (2001). Antioxidant Activity of Grape Seed (*Vitis vinifera*) Extracts on Peroxidation Models in Vitro. Food Chem..

[32] Yen G.-C., Chen H.-Y. (1995). Antioxidant Activity of Various Tea Extracts in Relation to Their Antimutagenicity. J. Agric. Food Chem..

[33] Braconi D., Bernardini G., Bianchini C., Laschi M., Millucci L., Amato L., Tinti L., Serchi T., Chellini F., Spreafico A. (2012). Biochemical and Proteomic Characterization of Alkaptonuric Chondrocytes. J. Cell. Physiol..

[34] Mosmann T. (1983). Rapid Colorimetric Assay for Cellular Growth and Survival: Application to Proliferation and Cytotoxicity Assays. J. Immunol. Methods.

[35] Filimonov D.A., Lagunin A.A., Gloriozova T.A., Rudik A.V., Druzhilovskii D.S., Pogodin P.V., Poroikov V.V. (2014). Prediction of the Biological Activity Spectra of Organic Compounds Using the Pass Online Web Resource. Chem. Heterocycl. Compd..

[36] Wishart D.S., Feunang Y.D., Guo A.C., Lo E.J., Marcu A., Grant J.R., Sajed T., Johnson D., Li C., Sayeeda Z. (2018). DrugBank 5.0: A Major Update to the DrugBank Database for 2018. Nucleic Acids Res..

[37] Bateman A., Martin M.-J., Orchard S., Magrane M., Ahmad S., Alpi E., Bowler-Barnett E., Britto R., Bye-A-Jee H., Cukura A. (2022). UniProt: The Universal Protein Knowledgebase in 2023. Nucleic Acids Res..

[38] Janson G., Paiardini A. (2021). PyMod 3: A complete suite for structural bioinformatics in PyMOL. Bioinformatics.

[39] Laskowski R.A., Rullmann J.A., MacArthur M.W., Kaptein R., Thornton J.M. (1996). AQUA and PROCHECK-NMR: Programs for Checking the Quality of Protein Structures Solved by NMR. J. Biomol. NMR.

[40] Laskowski R.A., MacArthur M.W., Moss D.S., Thornton J.M. (1993). PROCHECK: A Program to Check the Stereochemical Quality of Protein Structures. J. Appl. Cryst..

[41] Berendsen H.J.C., van der Spoel D., van Drunen R. (1995). GROMACS: A Message-Passing Parallel Molecular Dynamics Implementation. Comput. Phys. Commun..

[42] Vanommeslaeghe K., Hatcher E., Acharya C., Kundu S., Zhong S., Shim J., Darian E., Guvench O., Lopes P., Vorobyov I. (2010). CHARMM General Force Field: A Force Field for Drug-Like Molecules Compatible with the CHARMM All-Atom Additive Biological Force Fields. J. Comput. Chem..

[43] Jo S., Kim T., Iyer V.G., Im W. (2008). CHARMM-GUI: A Web-Based Graphical User Interface for CHARMM. J. Comput. Chem..

[44] Kim S., Chen J., Cheng T., Gindulyte A., He J., He S., Li Q., Shoemaker B.A., Thiessen P.A., Yu B. (2019). PubChem 2019 Update: Improved Access to Chemical Data. Nucleic Acids Res..

[45] Garullo G., Saponara S., Ahmed A., Gorelli B., Mazzotta S., Trezza A., Gianibbi B., Campiani G., Fusi F., Aiello F. (2022). Novel Labdane Diterpenes-Based Synthetic Derivatives: Identification of a Bifunctional Vasodilator That Inhibits CaV1.2 and Stimulates KCa1.1 Channels. Mar. Drugs.

[46] Rosignoli S., Paiardini A. (2022). DockingPie: A Consensus Docking Plugin for PyMOL. Bioinformatics.

[47] Morris G.M., Huey R., Lindstrom W., Sanner M.F., Belew R.K., Goodsell D.S., Olson A.J. (2009). AutoDock4 and AutoDockTools4: Automated Docking with Selective Receptor Flexibility. J. Comput. Chem..

[48] Trott O., Olson A.J. (2010). AutoDock Vina: Improving the Speed and Accuracy of Docking with a New Scoring Function, Efficient Optimization, and Multithreading. J. Comput. Chem..

[49] O’Boyle N.M., Banck M., James C.A., Morley C., Vandermeersch T., Hutchison G.R. (2011). Open Babel: An Open Chemical Toolbox. J. Cheminform..

[50] Salentin S., Schreiber S., Haupt V.J., Adasme M.F., Schroeder M. (2015). PLIP: Fully Automated Protein-Ligand Interaction Profiler. Nucleic Acids Res..

[51] Thompson J.D., Higgins D.G., Gibson T.J. (1994). CLUSTAL W: Improving the Sensitivity of Progressive Multiple Sequence Alignment through Sequence Weighting, Position-Specific Gap Penalties and Weight Matrix Choice. Nucleic Acids Res..

[52] Sievers F., Wilm A., Dineen D., Gibson T.J., Karplus K., Li W., Lopez R., McWilliam H., Remmert M., Söding J. (2011). Fast, Scalable Generation of High-Quality Protein Multiple Sequence Alignments Using Clustal Omega. Mol. Syst. Biol..

[53] Rudrapal M., Khairnar S.J., Khan J., Dukhyil A.B., Ansari M.A., Alomary M.N., Alshabrmi F.M., Palai S., Deb P.K., Devi R. (2022). Dietary Polyphenols and Their Role in Oxidative Stress-Induced Human Diseases: Insights into Protective Effects, Antioxidant Potentials and Mechanism(s) of Action. Front. Pharmacol..

[54] Gassel M., Breitenlechner C.B., Rüger P., Jucknischke U., Schneider T., Huber R., Bossemeyer D., Engh R.A. (2003). Mutants of Protein Kinase A That Mimic the ATP-Binding Site of Protein Kinase B (AKT). J. Mol. Biol..

[55] Fares M., Eldehna W.M., Bua S., Lanzi C., Lucarini L., Masini E., Peat T.S., Abdel-Aziz H.A., Nocentini A., Keller P.A. (2020). Discovery of Potent Dual-Tailed Benzenesulfonamide Inhibitors of Human Carbonic Anhydrases Implicated in Glaucoma and in Vivo Profiling of Their Intraocular Pressure-Lowering Action. J. Med. Chem..

[56] Song F.-L., Gan R.-Y., Zhang Y., Xiao Q., Kuang L., Li H.-B. (2010). Total Phenolic Contents and Antioxidant Capacities of Selected Chinese Medicinal Plants. Int. J. Mol. Sci..

[57] Eliopoulos C., Markou G., Langousi I., Arapoglou D. (2022). Reintegration of Food Industry By-Products: Potential Applications. Foods.

[58] Guine R., Costa C., Florença S., Correia P. (2023). A Review of the Use of Chestnut in Traditional and Innovative Food Products. J. Nuts.

[59] Rațu R.N., Veleșcu I.D., Stoica F., Usturoi A., Arsenoaia V.N., Crivei I.C., Postolache A.N., Lipșa F.D., Filipov F., Florea A.M. (2023). Application of Agri-Food By-Products in the Food Industry. Agriculture.

[60] Salvador R., Barros M.V., Donner M., Brito P., Halog A., De Francisco A.C. (2022). How to Advance Regional Circular Bioeconomy Systems? Identifying Barriers, Challenges, Drivers, and Opportunities. Sustain. Prod. Consum..

[61] Vannacci G., Bellini E. Chestnut (*Castanea sativa*): A Multipurpose European Tree. Proceedings of the Workshop.

[62] Conedera M., Krebs P., Tinner W., Pradella M., Torriani D. (2004). The Cultivation of *Castanea sativa* (Mill.) in Europe, from Its Origin to Its Diffusion on a Continental Scale. Veg. Hist. Archaeobot..

[63] Frusciante L., Geminiani M., Olmastroni T., Mastroeni P., Trezza A., Salvini L., Lamponi S., Spiga O., Santucci A. (2024). Repurposing *Castanea sativa* Spiny Burr By-Products Extract as a Potentially Effective Anti-Inflammatory Agent for Novel Future Biotechnological Applications. Life.

[64] Pal P., Singh A.K., Srivastava R.K., Rathore S.S., Sahoo U.K., Subudhi S., Sarangi P.K., Prus P. (2024). Circular Bioeconomy in Action: Transforming Food Wastes into Renewable Food Resources. Foods.

[65] Munteanu I.G., Apetrei C. (2021). Analytical Methods Used in Determining Antioxidant Activity: A Review. Int. J. Mol. Sci..

[66] Rumpf J., Burger R., Schulze M. (2023). Statistical Evaluation of DPPH, ABTS, FRAP, and Folin-Ciocalteu Assays to Assess the Antioxidant Capacity of Lignins. Int. J. Biol. Macromol..

[67] Sethi S., Joshi A., Arora B., Sharma R. (2020). Significance of FRAP, DPPH, and CUPRAC Assays for Antioxidant Activity Determination in Apple Fruit Extracts. Eur. Food Res. Technol..

[68] Sunjic S.B., Gasparovic A.C., Jaganjac M., Rechberger G., Meinitzer A., Grune T., Kohlwein S.D., Mihaljevic B., Zarkovic N. (2021). Sensitivity of Osteosarcoma Cells to Concentration-Dependent Bioactivities of Lipid Peroxidation Product 4-Hydroxynonenal Depend on Their Level of Differentiation. Cells.

[69] Sadowska-Bartosz I., Bartosz G. (2020). Effect of Antioxidants on the Fibroblast Replicative Lifespan In Vitro. Oxid. Med. Cell. Longev..

[70] Yudoh K., van Trieu N., Nakamura H., Hongo-Masuko K., Kato T., Nishioka K. (2005). Potential Involvement of Oxidative Stress in Cartilage Senescence and Development of Osteoarthritis: Oxidative Stress Induces Chondrocyte Telomere Instability and Downregulation of Chondrocyte Function. Arthritis Res. Ther..

